# Frequency-Stratified Changes in BDNF, IGF-1, and Cognitive Screening Scores Following a 16-Week Hatha Yoga Program in Older Women: A Quasi-Experimental Study

**DOI:** 10.3390/healthcare14081012

**Published:** 2026-04-12

**Authors:** Seonyoung Son, Suhan Koh, Taehyung Kim, Minkyo Kim, Daniel Newmire, Taekyu Kim, Doyeon Kim

**Affiliations:** 1 Exercise Physiology Laboratory, Department of Physical Education, Pusan National University, Busan 46241, Republic of Korea; 2 School of Health Promotion and Kinesiology, Texas Women’s University, Denton, TX 76204, USA

**Keywords:** Hatha Yoga, older women, neurotrophic factors, BDNF, IGF-1, cognitive function

## Abstract

**Highlights:**

**What are the main findings?**

- Changes in serum BDNF and IGF-1 were observed in older women through a 16-week Hatha Yoga program.
- Cognitive screening scores (CIST) showed small changes over time, and these findings should be interpreted cautiously as screening-level reference data.

**What are the implications of the main findings?**

- Hatha Yoga is considered a safe, low- to moderate-intensity activity for older women, and changes in peripheral BDNF and IGF-1 can be reported as reference data in this population.
- The effects of practice frequency were not conclusive in this quasi-experimental study, particularly where group × time interactions were not significant. Therefore, the findings can be used as preliminary reference data for future randomized or well-controlled studies.

**Abstract:**

**Background/Objectives:** Aging is associated with declines in cognitive function and neurotrophic support. Brain-derived neurotrophic factor (BDNF) and insulin-like growth factor-1 (IGF-1) are peripheral biomarkers discussed in relation to brain health and aging. This study investigated changes in serum BDNF, IGF-1, and cognitive screening scores after a 16-week Hatha Yoga program performed twice or four times per week in older women. **Methods:** Fifty-one community-dwelling women aged 70–79 years were allocated to a twice-per-week yoga group (2YG; *n* = 17), a four-times-per-week yoga group (4YG; *n* = 17), or a non-exercise control group (CON; *n* = 17) based on availability and participant preference; forty-three participants completed the study. Serum BDNF and IGF-1 were analyzed using enzyme-linked immunosorbent assay and chemiluminescent immunoassay, and cognitive status was evaluated using the Cognitive Impairment Screening Test (CIST). Outcomes were analyzed using two-way repeated-measures ANOVA and additional ANCOVA models adjusting for corresponding baseline values. Exploratory correlations were examined between biomarker changes and CIST changes. Effect sizes and 95% confidence intervals were reported. **Results:** BDNF showed a significant main effect of time (*p* < 0.05) without a significant group × time interaction; ANCOVA adjusting for baseline BDNF showed no significant group effect (*p* = 0.270). IGF-1 showed a significant group × time interaction (*p* < 0.01) with increases in both yoga groups; ANCOVA adjusting for baseline IGF-1 showed a significant group effect (*p* = 0.001). CIST showed a significant main effect of time (*p* < 0.01), but changes were small and the group × time interaction was not significant; ANCOVA adjusting for baseline CIST showed no significant group effect (*p* = 0.114). Biomarker changes were not clearly correlated with CIST changes (ΔBDNF–ΔCIST: r = −0.244, *p* = 0.115; ΔIGF-1–ΔCIST: r = −0.050, *p* = 0.750). **Conclusions:** In this quasi-experimental study with non-random allocation and limited covariate information, changes in peripheral neurotrophic factors and only small changes in cognitive screening scores were observed after participation in a 16-week Hatha Yoga program. However, frequency-dependent conclusions are limited, and findings should be interpreted cautiously as screening-level, hypothesis-generating reference data. Nevertheless, the program is considered a feasible, low-risk health promotion activity for older women and may inform future randomized or well-controlled studies.

## 1. Introduction

The global demographic shift toward an aging society is accelerating, with South Korea emerging as one of the fastest-aging nations. As of late 2024, over 20% of the population was aged 65 years or older, officially classifying the country as a super-aged society [1]. This demographic transition is accompanied by a sharp increase in age-related health challenges, particularly neurodegenerative diseases such as Alzheimer’s disease. Cognitive decline among older adults not only undermines their quality of life but also imposes substantial socioeconomic burdens on healthcare systems [2]. Consequently, identifying effective strategies to delay or mitigate cognitive impairment in older persons is a public health and scientific priority.

Age-related cognitive decline is closely linked to structural and functional changes in the brain including hippocampal and prefrontal atrophy, synaptic loss, and neuronal degeneration [3]. Among the neurobiological mechanisms implicated, reductions in brain-derived neurotrophic factor (BDNF) and insulin-like growth factor-1 (IGF-1) play critical roles. BDNF is a key protein involved in neuronal survival, synaptic plasticity, and memory enhancement. Its levels naturally decline with age and are associated with decreased hippocampal volume and impaired memory function [4]. Notably, patients with Alzheimer’s disease exhibit significantly lower levels of BDNF than cognitively healthy older adults, making BDNF a potential biomarker for neurodegenerative disorders [5].

IGF-1 has been discussed as a growth-related factor that may be related to brain health and aging, although peripheral concentrations do not directly indicate central mechanisms [6]. Age-related changes in IGF-1 have been reported to be associated with neurological function and cognitive outcomes in older adults [7], but these relationships are complex and require caution in interpretation. Previous studies have consistently reported that low BDNF and IGF-1 levels are significantly correlated with reduced cognitive performance and elevated dementia risk [8,9].

Importantly, both BDNF and IGF-1 levels appear to be modifiable through physical activity. Exercise interventions have been reported to be associated with changes in BDNF and IGF-1 in older adults, but the magnitude of change and its relation to cognitive outcomes may differ across study designs and populations [10,11]. However, most previous studies have implemented aerobic and high-intensity exercises to improve BDNF and IGF-1 [12,13].

Among various exercise modalities, Hatha Yoga—a mind–body practice that combines asanas (physical postures), pranayama (breathing techniques), and meditation—has gained attention as a feasible option for older adults. Hatha Yoga practice has been associated with improvements in muscular strength, flexibility, balance, and cardiovascular function, as well as psychological outcomes such as stress reduction; therefore, it is considered a practical form of physical activity that may be related to cognitive health in older adults [14,15].

Additionally, the study by Küçük et al. [16] presented the acute and chronic effects of high-intensity exercise, but it poses risks for older women to perform. Therefore, despite the growing interest in yoga-based interventions, existing studies have primarily examined BDNF, IGF-1, and cognitive function individually, and research exploring the relationships among these variables from an integrative perspective is very limited. Furthermore, little is known about the effects of moderate-intensity exercise frequency on the neurobiological and cognitive responses in older adults.

**Figure 1:**
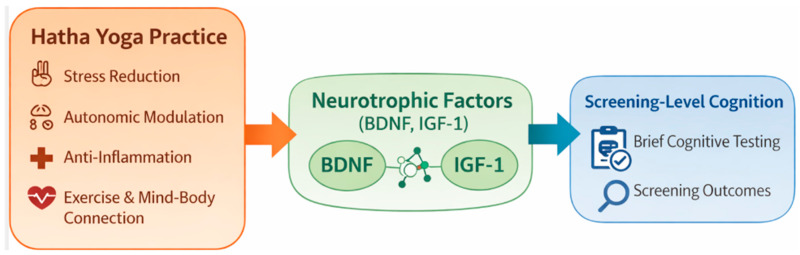
Conceptual framework summarizing hypothesis-generating pathways linking Hatha Yoga practice to peripheral biomarkers (BDNF, IGF-1) and screening-level cognition. The proposed pathways are illustrative and were not directly tested in this study.

From an integrative perspective, Hatha Yoga may be associated with peripheral neurotrophic factors and screening-level cognitive outcomes through multiple pathways, such as stress reduction and autonomic modulation, mind–body regulation, and the physical activity component involving repeated muscle activation. Figure 1 presents a conceptual framework to summarize these hypothesis-generating pathways, recognizing that the present study does not directly test mechanistic links.

This study aimed to examine whether a 16-week Hatha Yoga program performed twice or four times per week is associated with changes in peripheral neurotrophic factors and cognitive screening scores in older women, while recognizing that the quasi-experimental design limits causal inference.

## 2. Materials and Methods

### 2.1. Participants

The sample size was estimated using G*Power 3.1 program (Heinrich-Heine University Düsseldorf, Düsseldorf, Germany) to ensure feasibility and detect medium effect sizes across the naturally occurring comparison groups in this quasi-experimental design. The parameters were set as follows: effect size (*f*) = 0.25 (medium), statistical power (1 − *β*) = 0.80, significance level (*α*) = 0.05, with three groups and two measurement time points. The minimum sample size was 42. Initially, 51 participants were recruited to account for potential dropouts.

Eligible participants were community-dwelling women aged 70 to 79 years residing in city M, who had not engaged in regular physical activity in the past three months and scored 24 or higher on the Cognitive Impairment Screening Test (CIST), since this was a human-subject study, participants were assigned to one of the three groups based on individual circumstances, situation, program availability, schedule flexibility, and participant preference, and only data from participants who attended at least 80% of the exercise sessions were included in the analysis. In non-random quasi-experimental studies where allocation is not random and groups are assigned based on availability or participants’ preferences, there is a risk of selection bias, so this serves as an exploratory, hypothesis-generating basic study rather than a confirmatory one.

- Yoga Group I: Hatha Yoga twice per week (*n* = 17)
- Yoga Group II: Hatha Yoga four times per week (*n* = 17)
- Control Group: No intervention (*n* = 17)

During the 16-week intervention period, eight participants withdrew because of personal circumstances or incomplete data. In Yoga Group I (*n* = 2), the dropout was due to unreliable blood test results, while in Yoga Group II (*n* = 2) and the control group (*n* = 4), participants withdrew for personal reasons (health issues, moving, or opting out). Ultimately, 43 participants were included in the final analysis, of which 15 were in the twice-per-week yoga group, 15 were in the four times per week yoga group, and 13 were in the control group.

This study was approved by the Institutional Review Board of Pusan National University (IRB No. PNU IRB/2024_34_HR). As this study did not involve pharmacological treatment or a randomized intervention, registration in a clinical trial registry was not required under national research guidelines. All participants provided written informed consent after receiving a full explanation of the study’s purpose and procedures. Descriptive statistics of the participants’ physical characteristics are presented in Table 1.

### 2.2. Measurements and Procedures

**Figure 2:**
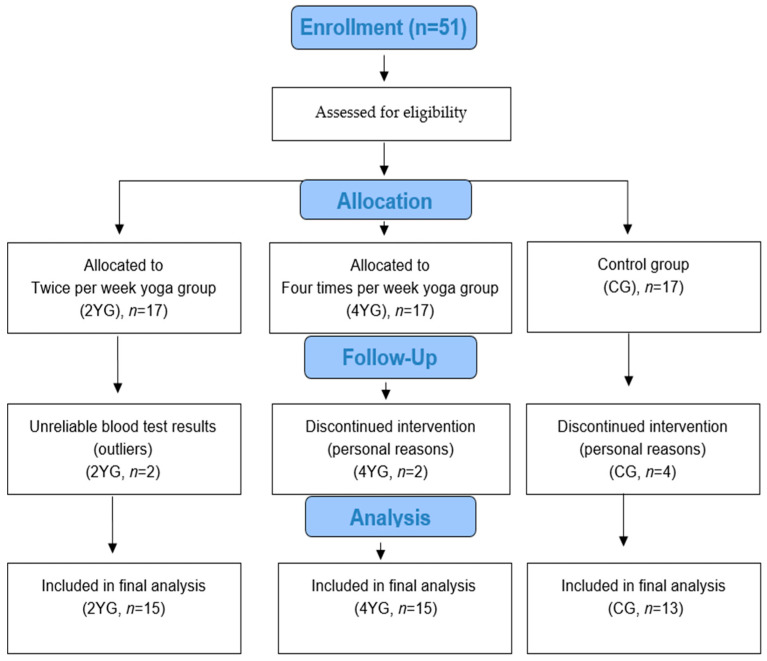
Flow diagram of the study.

Before data collection, all participants were fully informed of the study’s purpose, procedures, and potential risks. Each participant completed a self-report questionnaire that assessed their general health status and physical activity history. All measurements were conducted under standardized conditions at two time points: pre-intervention (baseline) and post-intervention (after 16 weeks). Figure 2 shows the participation flowchart.

#### 2.2.1. Body Composition

Body composition, namely height, weight, body mass index, and body fat percentage, was assessed using a bioelectrical impedance analyzer (InBody 430, Biospace, Seoul, Republic of Korea). Measurements were taken with the participants wearing light clothing and without shoes.

#### 2.2.2. Cognitive Screening Scores

Cognitive screening scores were evaluated using the CIST, a validated tool developed by the Korean Ministry of Health and Welfare for the National Dementia Screening Program. The CIST comprises six domains—memory, language, orientation, attention, visuospatial ability, and executive function—with a possible total score of 30. A score of ≥24 indicates normal cognition, 20–23 suggests mild cognitive impairment, and ≤19 indicates possible dementia. Only participants who scored 24 or higher at baseline were included in the study.

### 2.3. Blood Sampling and Biochemical Analyses

Participants were instructed to fast for at least 8 h before blood collection, beginning at 9:00 p.m. the evening before blood collection. Blood samples were collected in the morning (e.g., 09:00–10:00) after an overnight fast to reduce diurnal variability. All samples were processed using the same protocol and analyzed in the same batch to reduce analytical variability.

Venous blood (10 mL) was drawn from the forearm into serum tubes (SST). The samples were centrifuged at 3000 rpm for 20 min using a refrigerated centrifuge (Combi-514R, Hanil, Seoul, Republic of Korea). The separated serum was aliquoted into 1.5 mL microtubes and stored at −80 °C until analysis.

Serum levels of BDNF were measured using a sandwich enzyme-linked immunosorbent assay. All reagents and standards were prepared at room temperature (21 °C), and absorbance was read at 450 nm using a microplate reader (SpectraMax190, Molecular Devices, San Jose, CA, USA).

Serum IGF-1 concentrations were analyzed using chemiluminescent immunoassay. Commercial assay kits (IGF-1, Roche, Mannheim, Germany) were used and analyses were performed using a COBAS e801 analyzer (Roche, Mannheim, Germany). The reference range for IGF-1 levels was set at 49.6–185.0 ng/mL.

### 2.4. Hatha Yoga Intervention

The Hatha Yoga program used in this study was adapted from the protocol developed by Yoon et al. (2015) [11] and modified to accommodate the physical characteristics of older women and the objectives of this study. The intervention lasted for 16 weeks, with participants attending either two or four sessions per week, depending on group allocation. Each session lasted 60 min, consisting of a 10 min warm-up, 40 min main practice, and 10 min cool-down period.

The program incorporated fundamental Hatha Yoga postures designed to promote flexibility, muscular strength, and cardiopulmonary function. Exercise intensity was calibrated using a pilot test involving five women in their 70s. Heart rate and subjective exertion levels were monitored using a heart rate monitor (Polar M400, Kempele, Finland) and a rating of perceived exertion (RPE) scale. Based on these findings, exercise intensity was maintained within an RPE range of 9–13 and 40–60% of heart rate reserve (HRR) to ensure safety and suitability for the population. Assessments of appropriate skills, safety, and continuous participation were made in all sessions, and adjustments to exercise intensity were carefully monitored under the supervision of the first author. Table 2 presents session content in detail.

### 2.5. Statistical Analysis

All statistical analyses were performed using SPSS version 27.0 (IBM Corp., Armonk, NY, USA). Descriptive statistics are presented as mean (M) and standard deviation (SD), and normality was assessed using the Shapiro–Wilk test. To examine changes over time across groups, a two-way repeated-measures ANOVA was conducted with group (2YG, 4YG, CON) as the between-subject factor and time (pre, post) as the within-subject factor. When significant effects were observed, Bonferroni-adjusted post hoc comparisons were used for pairwise group comparisons. Baseline equivalence of outcome variables (BDNF, IGF-1, and CIST) was examined using one-way ANOVA at pre-intervention, and standardized mean differences were calculated (Table S1).

In addition, ANCOVA models were performed to evaluate group differences at post-intervention while adjusting for the corresponding baseline value (post as the dependent variable, group as the fixed factor, and baseline as a covariate). Exploratory Pearson correlation analyses were conducted to examine associations between changes in biomarkers and changes in CIST scores (ΔBDNF ↔ ΔCIST; ΔIGF-1 ↔ ΔCIST).

In addition to *p*-values, effect sizes were reported to aid interpretation: partial eta-squared (ηp^2^) was reported for ANOVA effects, and standardized within-group pre–post changes were summarized using Hedges’ g with 95% confidence intervals (obtained using bootstrap resampling). Partial η^2^ magnitudes were interpreted using conventional guidelines (small ≈ 0.01, medium ≈ 0.06, large ≈ 0.14). The statistical significance level was set at α = 0.05.

## 3. Results

Effect sizes with 95% confidence intervals are presented in Table 3, Table 4 and Table 5 to aid interpretation of the magnitude of changes.

### 3.1. Changes in Serum BDNF Levels

**Figure 3:**
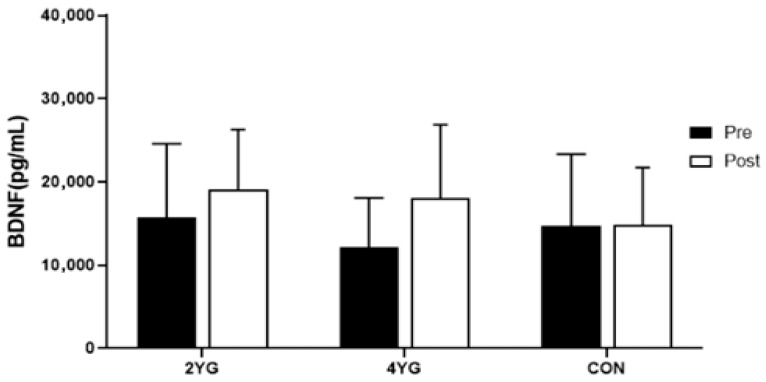
Comparisons of BDNF after 16-week yoga exercise. 2YG: twice per week yoga group, 4YG: four times per week yoga group, CON: control group.

Table 3 and Figure 3 present the serum BDNF levels across groups. A two-way repeated measures ANOVA revealed a significant main effect of time (F(1,40) = 5.284, *p* = 0.027, partial η^2^ = 0.117), whereas the group × time interaction (F(2,40) = 1.471, *p* = 0.242, partial η^2^ = 0.069) and the main effect of group (F(2,40) = 0.784, *p* = 0.463, partial η^2^ = 0.038) were not statistically significant. BDNF increased from pre- to post-intervention in both yoga groups (2YG: 15,806.67 to 19,120.00 pg/mL; 4YG: 12,093.53 to 18,073.33 pg/mL), whereas only a small change was observed in the control group (14,669.23 to 14,823.08 pg/mL).

**Table 3:** Changes in BDNF after 16-week Hatha Yoga exercise program.

Variable	Group	Pre	Post	Δ (Post-Pre)	Hedges’ g (95% CI)
BDNF (pg/mL)	2YG (*n* = 15)	15,806.67 ± 8778.99	19,120.00 ± 7159.03	3313.33	0.36 (−0.12, 0.84)
	4YG (*n* = 15)	12,093.53 ± 5993.78	18,073.33 ± 8794.84	5979.80	0.65 (0.16, 1.79)
	CON (*n* = 13)	14,669.23 ± 8667.22	14,823.08 ± 6876.31	153.85	0.02 (−0.54, 0.59)
	*Bonferroni* (post hoc)	*NS*	*NS*		

Values are presented as mean ± SD. 2YG: twice per week Hatha Yoga group; 4YG: four times per week Hatha Yoga group; CON: control group. Δ indicates Post–Pre. Hedges’ g (95% CI) represents the standardized within-group pre–post change (small-sample corrected); 95% confidence intervals were obtained using bootstrap resampling. Bonferroni post hoc test was performed for pairwise comparisons. NS, not significant.

Standardized within-group pre–post changes are summarized in Table 3 using Hedges’ g with 95% confidence intervals. These findings indicate that BDNF levels changed over time across participants regardless of group. However, because the interaction effect was not significant, differences according to practice frequency cannot be concluded, and the within-group changes should be interpreted with caution. In ANCOVA adjusting for baseline BDNF, the group effect on post-intervention BDNF was not statistically significant (*p* = 0.270). The baseline value was a significant covariate (*p* = 0.031).

### 3.2. Changes in Serum IGF-1 Levels

**Figure 4:**
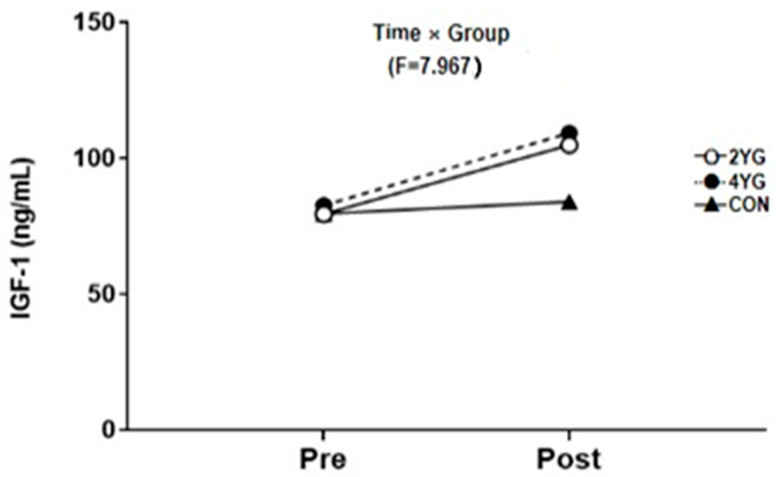
Comparisons of two-way repeated measures ANOVA of IGF-1 after 16-week yoga exercise. 2YG: twice per week yoga group, 4YG: four times per week yoga group, CON: control group.

**Figure 5:**
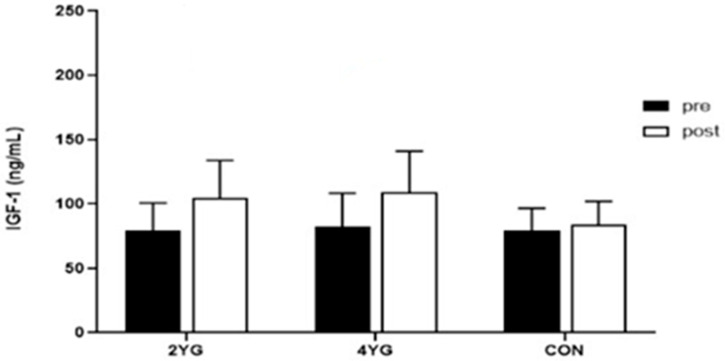
Comparisons of IGF-1 after 16-week Hatha Yoga exercise. 2YG: twice per week yoga group, 4YG: four times per week yoga group, CON: control group.

Table 4 and Figure 4 and Figure 5 present serum IGF-1 levels across groups. A two-way repeated measures ANOVA revealed a significant group × time interaction (F(2,40) = 7.967, *p* < 0.001, partial η^2^ = 0.300) and a significant main effect of time (F(1,40) = 56.730, *p* < 0.001, partial η^2^ = 0.601), whereas the main effect of group was not statistically significant (F(2,40) = 1.416, *p* = 0.255, partial η^2^ = 0.066). IGF-1 increased from pre- to post-intervention in both yoga groups (2YG: 79.29 ± 21.37 to 104.73 ± 29.11 ng/mL; 4YG: 82.42 ± 25.77 to 109.79 ± 30.75 ng/mL), whereas only a small change was observed in the control group (79.43 ± 17.18 to 83.83 ± 18.20 ng/mL).

**Table 4:** Changes in IGF-1 after 16-week Hatha Yoga exercise program.

Variable	Group	Pre	Post	Δ (Post-Pre)	Hedges’ g (95% CI)
IGF-1 (ng/mL)	2YG (*n* = 15)	79.29 ± 21.37	104.73 ± 29.11	25.44	1.61 (1.21, 2.50)
	4YG (*n* = 15)	82.42 ± 25.77	109.79 ± 30.75	27.37	1.43 (0.80, 3.41)
	CON (*n* = 13)	79.43 ± 17.18	83.83 ± 18.20	4.40	0.28 (−0.27, 0.85)
	*Bonferroni* (post hoc)	*NS*	*NS*		

Values are presented as mean ± SD. 2YG: twice per week Hatha Yoga group; 4YG: four times per week Hatha Yoga group; CON: control group. Δ indicates Post–Pre. Hedges’ g (95% CI) represents the standardized within-group pre–post change (small-sample corrected); 95% confidence intervals were obtained using bootstrap resampling. Bonferroni post hoc test was performed for pairwise comparisons. NS, not significant.

Standardized within-group pre–post changes are summarized in Table 4 using Hedges’ g with 95% confidence intervals. Bonferroni-adjusted post hoc comparisons did not reveal statistically significant between-group differences at either the pre- or post-intervention time points. In ANCOVA adjusting for baseline IGF-1, the group effect on post-intervention IGF-1 remained statistically significant (*p* = 0.001). However, given the non-random allocation and limited covariate information, these findings require caution in interpretation and cannot be taken as evidence of causal frequency-specific effects.

### 3.3. Changes in Cognitive Function (CIST Scores)

**Figure 6:**
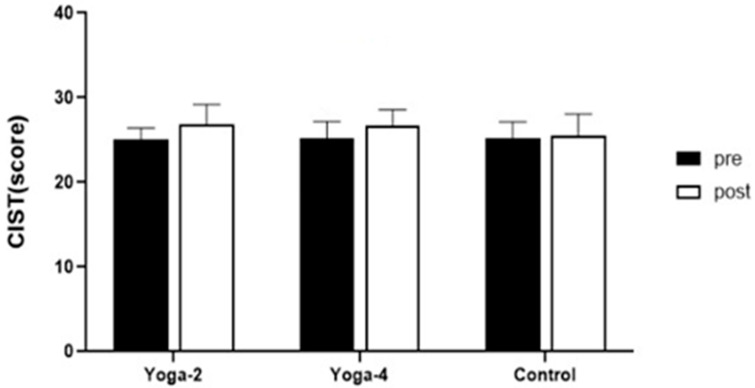
Comparisons of CIST after 16-week Hatha Yoga exercise. 2YG: twice per week Hatha Yoga group, 4YG: four times per week Hatha Yoga group, CON: control group.

Table 5 and Figure 6 present CIST scores across groups. A two-way repeated measures ANOVA revealed a significant main effect of time (F(1,40) = 19.330, *p* < 0.001, partial η^2^ = 0.326), whereas the group × time interaction (F(2,40) = 2.283, *p* = 0.115, partial η^2^ = 0.102) and the main effect of group (F(2,40) = 0.503, *p* = 0.609, partial η^2^ = 0.025) were not statistically significant. CIST scores increased from pre- to post-intervention in both yoga groups (2YG: 25.07 to 26.87; 4YG: 25.20 to 26.67), whereas only a small change was observed in the control group (25.15 to 25.54).

**Table 5:** Changes in CIST after 16-week Hatha Yoga exercise program.

Variable	Group	Pre	Post	Δ (Post-Pre)	Hedges’ g (95% CI)
CIST (score)	2YG (*n* = 15)	25.07 ± 1.33	26.87 ± 2.33	1.80	0.88 (0.48, 1.52)
	4YG (*n* = 15)	25.20 ± 1.97	26.67 ± 1.91	1.47	0.92 (0.64, 1.46)
	CON (*n*=13)	25.15 ± 1.99	25.54 ± 2.50	0.38	0.18 (−0.48, 0.57)
	*Bonferroni* (post hoc)	*NS*	*NS*		

Values are presented as mean ± SD. 2YG: twice per week Hatha Yoga group; 4YG: four times per week Hatha Yoga group; CON: control group. Δ indicates Post–Pre. Hedges’ g (95% CI) represents the standardized within-group pre–post change (small-sample corrected); 95% confidence intervals were obtained using bootstrap resampling. Bonferroni post hoc test was performed for pairwise comparisons. NS, not significant.

Standardized within-group pre–post changes are summarized in Table 5 using Hedges’ g with 95% confidence intervals. Because the interaction effect was not significant and the magnitude of change was small, these results are best interpreted as modest changes in cognitive screening scores over time, and differences according to practice frequency cannot be concluded. Given that CIST is a brief screening tool, the cognitive findings should be interpreted as screening-level results rather than evidence of cognitive improvement. In ANCOVA adjusting for baseline CIST, the group effect on post-intervention CIST was not statistically significant (*p* = 0.114).

### 3.4. Exploratory Associations Between Biomarker Changes and CIST Changes

Exploratory Pearson correlation analyses did not show a clear association between changes in serum BDNF and changes in CIST scores (r = −0.244, *p* = 0.115), or between changes in IGF-1 and changes in CIST scores (r = −0.053, *p* = 0.738). These findings suggest that peripheral biomarker changes did not translate directly to detectable changes in screening-level cognition over the intervention period, and the results require caution in interpretation.

## 4. Discussion

### 4.1. Changes in BDNF Levels

BDNF is associated with neuronal survival, synaptic plasticity, and cognitive function, and generally follows a circadian rhythm, decreasing during sleep and increasing while awake, and is greatly influenced by physical activity [17]. Various forms of regular exercise have been reported to increase blood BDNF levels [18,19]; therefore, in this study, Hatha Yoga activities combining dynamic movements, isometric strength, and breath control are considered to provide stimulation that may be associated with changes in blood BDNF levels.

The results of this study showed a tendency for blood BDNF levels to increase after 16 weeks of Hatha Yoga practice, but since only a main effect over time existed, changes according to the frequency of yoga sessions are not conclusive. In particular, the participants in the group practicing Hatha Yoga four times a week had mean blood BDNF levels that, although not statistically significant, were lower in the initial test compared to other groups. Previous studies [20,21] reported that individuals with initially low BDNF levels showed greater increases after exercise, which could indicate a kind of ceiling effect and thus requires caution in interpreting the results.

However, in the study by Kang et al. [22], it was reported that BDNF levels increased after performing aquatic exercise at 50–70% HRR three times per week for 16 weeks. This may support the results of the present study, which showed a tendency for BDNF to increase following moderate-intensity Hatha Yoga. Regular physical activity has been associated with higher peripheral BDNF levels in previous studies. However, in the present study, BDNF showed a significant main effect of time without a significant group × time interaction, and peripheral BDNF levels do not allow conclusions about central neuroplasticity or protection against cognitive decline. Therefore, future research is recommended to use this as a baseline to explore various intensities and durations, as well as to conduct central neurogenesis assessments or neuroimaging examinations.

Nevertheless, moderate-intensity Hatha Yoga performed by older women who do not engage in regular exercise is considered suitable for them, as it carries lower physical risk than high-intensity exercise and can be practiced without being constrained by location.

### 4.2. Changes in IGF-1 Levels

IGF-1 has been discussed as a growth-related factor that may be related to neuronal maintenance and cognitive function, and in the present study, it increased in both yoga groups after the 16-week Hatha Yoga program. The Hatha Yoga performed in this study consisted of a combination of static and dynamic movements, and repeated muscle tension and relaxation during stretching and holding postures may have been associated with the observed increase in IGF-1 [23].

IGF-1 increased over time in both yoga groups, and a significant group × time interaction was observed. However, because the groups were formed without random allocation and potential confounders were not statistically adjusted, the between-group pattern requires caution in interpretation, and differences according to practice frequency cannot be concluded. In addition, IGF-1 was measured in peripheral blood only, and no neuroimaging or other direct assessments of central mechanisms were included. Therefore, mechanistic interpretations such as neurogenesis or neuroprotection should be avoided, and future controlled studies are recommended to clarify whether and how yoga-related activity is associated with IGF-1 changes and related outcomes.

### 4.3. Cognitive Function

Cognitive functions involve complex mental processes such as memory, attention, language, and executive functions, and are primarily regulated by the frontal lobe and hippocampus [24,25]. Aging is associated with a decrease in neuron density and connectivity in these areas, which can lead to cognitive decline. However, in the case of mild cognitive impairment, recovery is often possible through appropriate interventions such as physical activity or cognitive training [26].

As a result of this study, a significant main effect over time was observed after a 16-week Hatha Yoga program, and both groups that performed Hatha Yoga showed a tendency for increased CIST scores in the rate of change. However, the improvement effect was minimal, and since these changes could reflect practice effects from previous tests, caution is needed in interpretation.

CIST is a brief global cognitive screening tool, and repeated testing may involve practice effects. In our study, CIST showed a significant main effect of time but no significant group × time interaction, and the magnitude of change was modest. Therefore, these findings are best interpreted as small changes in screening scores rather than evidence of cognitive enhancement. Future studies are recommended to use domain-specific neuropsychological assessments and longer follow-up to better evaluate cognitive outcomes.

Several reasons may explain why the observed biomarker changes did not translate into measurable changes in CIST scores. First, CIST is a brief screening tool and may be insufficiently sensitive to detect subtle domain-specific cognitive changes, and repeated testing may involve practice effects. Second, the magnitude of peripheral biomarker changes may have been too small, or the intervention duration may have been insufficient, to yield detectable cognitive changes. Third, peripheral biomarker changes do not necessarily reflect central mechanisms. Therefore, longer follow-up and domain-specific neuropsychological assessments are recommended in future controlled studies.

Taken together, Hatha Yoga may be a feasible multimodal activity for older women; however, the present findings should be considered exploratory and primarily informative for the design of future confirmatory studies.

### 4.4. Practical Implications and Limitations

This study provides preliminary data on changes in peripheral biomarkers and cognitive screening scores observed after participation in a 16-week Hatha Yoga program in older women.

First, since the intervention period was limited to 16 weeks and the sample size was small, it is difficult to draw clear conclusions regarding neurophysiological benefits and sustainability.

Second, cognitive assessment was not conducted for each cognitive domain, and it was evaluated using only the CIST, limiting the interpretability of cognitive changes.

Third, since measurements of the central nervous system or neurotrophic tests were not included and only peripheral biomarkers (BDNF, IGF-1) were analyzed, only indirect analysis was possible.

Finally, confounding variables (sleep, diet, medication use, social interaction, stress) were not fully controlled, and although the timing of blood sample collection was appropriate, for variables related to circadian rhythms, such as BDNF, the timing of blood collection needs to be considered.

Because detailed covariate information was not available in the present dataset, more advanced approaches such as propensity-score methods or multivariable confounder adjustment and sensitivity analyses could not be performed.

## 5. Conclusions

In this quasi-experimental study, participants were allocated without randomization, and no statistical adjustment was made for potential confounders. We observed time-related changes in serum IGF-1 and modest changes in cognitive screening scores, while BDNF showed a significant main effect of time without a significant group × time interaction. Because these design features limit causal inference, and cognition was assessed using a single screening measure, conclusions regarding cognitive enhancement or frequency-dependent efficacy require caution. Overall, these results can be used as preliminary data and may help inform future randomized or well-controlled studies with domain-specific cognitive assessments and additional measures to clarify underlying mechanisms.

## Figures and Tables

**Table 1:** Physical characteristics of participants in each group.

	Variables	Age (yrs)	Height (cm)	Weight (kg)	BMI (kg/m^2^)	%BF (%)
Group						
2YG (*n* = 15)		75.60 ± 3.20	152.4 ± 4.76	56.67 ± 4.15	24.44 ± 2.02	35.45 ± 3.81
4YG (*n* = 15)		74.87 ± 3.40	150.73 ± 4.27	54.30 ± 5.01	23.85 ± 1.87	32.67 ± 3.84
CON (*n* = 13)		74.62 ± 3.07	152.85 ± 4.69	55.35 ± 8.66	23.84 ± 2.49	34.2 ± 5.11

Values are *M ± SD.* BMI: body mass index, %BF: percentage of body fat, 2YG: twice per week Hatha Yoga group, 4YG: four times per week Hatha Yoga group, CON: control group.

**Table 2:** Hatha Yoga exercise program.

Section	Exercise	Intensity	Frequency
Warm-up (10 min)	Stretching		
Hatha Yoga (40 min)	Paschimottanasana Baddha Konasana Janu Sirsasan Upavistha Konasana Gomukhasana Urdhva Dhanurasana	40–50% HRR (RPE 9–11)	2 times, 4 times/ a week
	Surya Namaskar Utthita Trikonasana Parivrtta Trikonasana Vrksasana Virabhadrasana Bhujangasana Marjaryasana Adho Mukha Svanasana	50–60% HRR (RPE 12–13)	
	Uttanasana Supta Matsyendrasana Setu Bandhasana	40–50% HRR (RPE 9–11)	
Cool-down (10 min)	Savasana		

## Data Availability

The data presented in this study are available on request from the corresponding author due to the dataset contains participants’ (older women’s) personal health information and sensitive demographic variables. Even after de-identification, there remains a risk of re-identification due to the small sample size and the combination of variables. In addition, data sharing for this study is permitted only within the scope specified by the Institutional Review Board (IRB) approval and the participants’ informed consent.

## Supplementary Materials

The following supporting information can be downloaded at: https://www.mdpi.com/article/10.3390/healthcare14081012/s1, Table S1: Baseline levels of outcome variables across groups.

## Abbreviations

The following abbreviations are used in this manuscript:

ANOVA	Analysis of variance
BDNF	Brain-derived neurotrophic factor
CIST	Cognitive Impairment Screening Test
HRR	Heart rate reserve
IGF-1	Insulin-like growth factor-1
SD	Standard deviation
