# Supplementary material for: Frequency-Stratified Changes in BDNF, IGF-1, and Cognitive Screening Scores Following a 16-Week Hatha Yoga Program in Older Women: A Quasi-Experimental Study
Source: Healthcare (Basel). 2026 Apr 12;14(8):1012. doi: 10.3390/healthcare14081012 (PMC13116418; doi:10.3390/healthcare14081012)
Supplement: Supplementary file 1 [file healthcare-14-01012-s001.zip › healthcare-4101835-supplementary.pdf]

Table S1. Baseline levels of outcome variables across groups.

Variable	2YG (n=15)	4YG (n=15)	CON (n=13)	p-value (one-way ANOVA)	Max ∣SMD∣
BDNF (pg/mL)	15806.67 ± 8778.99	12093.53 ± 5993.79	14669.23 ± 8667.22	0.427	0.49
IGF-1 (ng/mL)	79.29 ± 21.37	82.42 ± 25.77	79.43 ± 17.18	0.909	0.13
CIST (score)	25.07 ± 1.33	25.20 ± 1.97	25.15 ± 1.99	0.979	0.08

Values are mean ± SD. p-values are from one-way ANOVA at baseline. SMD indicates standardized mean difference (absolute value).
